# Supplementary material for: SNP-based assessment of genetic purity and diversity in maize hybrid breeding
Source: PLoS One. 2021 Aug 3;16(8):e0249505. doi: 10.1371/journal.pone.0249505 (PMC8330893; doi:10.1371/journal.pone.0249505)
Supplement: S1 Table — (DOCX) [file pone.0249505.s001.docx]

**S1 Table. List of 30 parental maize lines and 158 experimental single-cross hybrids sourced from ARC-GCI**

| No | Name | Type | Pedigree | No | Name | Type | Pedigree |
| --- | --- | --- | --- | --- | --- | --- | --- |
| 1 | CB322 | Inbred line Inbred line lllllline Line |  | 95 | SCHP67 | Hybrid | CML442 / CML488 |
| 2 | CB323 | Inbred line |  | 96 | SCHP68 | Hybrid | CML442 / CML511 |
| 3 | CB339 | Inbred line |  | 97 | SCHP69 | Hybrid | CML442 / CML544 |
| 4 | CK21 | Inbred line |  | 98 | SCHP70 | Hybrid | CML442 / CML547 |
| 5 | CKDHL0089* | DH line |  | 99 | SCHP71 | Hybrid | CML442 / CZL068 |
| 6 | CKDHL0295* | DH line |  | 100 | SCHP72 | Hybrid | CML443 / CK21 |
| 7 | CKDHL0378* | DH line |  | 101 | SCHP73 | Hybrid | CML443/CML442 |
| 8 | CKDHL0470* | DH line |  | 102 | SCHP74 | Hybrid | CML443 / I-38 |
| 9 | CKL05022 | Inbred line |  | 103 | SCHP75 | Hybrid | CML443 / I-42 |
| 10 | CML202 | Inbred line |  | 104 | SCHP76 | Hybrid | CML444 / CK21 |
| 11 | CML216 | Inbred line |  | 105 | SCHP77 | Hybrid | CML444 / CML442 |
| 12 | CML442 | Inbred line |  | 106 | SCHP78 | Hybrid | CML444 / I-38 |
| 13 | CML443 | Inbred line |  | 107 | SCHP79 | Hybrid | CML444 / I-42 |
| 14 | CML444 | Inbred line |  | 108 | SCHP80 | Hybrid | CML488 / CK21 |
| 15 | CML488 | Inbred line |  | 109 | SCHP81 | Hybrid | CML488 / CML442 |
| 16 | CML511 | Inbred line |  | 110 | SCHP82 | Hybrid | CML488 / I-38 |
| 17 | CML543 | Inbred line |  | 111 | SCHP83 | Hybrid | CML488 / RO549W |
| 18 | CML544 | Inbred line |  | 112 | SCHP84 | Hybrid | CML488 / U2540W |
| 19 | CML547 | Inbred line |  | 113 | SCHP85 | Hybrid | CML511 / CK21 |
| 20 | CZL068 | Inbred line |  | 114 | SCHP86 | Hybrid | CML511 / CML442 |
| 21 | I-38 | Inbred line |  | 115 | SCHP87 | Hybrid | CML511 / I-38 |
| 22 | I-42 | Inbred line |  | 116 | SCHP88 | Hybrid | CML511 / I-42 |
| 23 | RO549W | Inbred line |  | 117 | SCHP89 | Hybrid | CML511 / RO549W |
| 24 | U2540W | Inbred line |  | 118 | SCHP90 | Hybrid | CML543 / I-38 |
| 25 | CML540 | Inbred line |  | 119 | SCHP91 | Hybrid | CML543 / RO549W |
| 26 | CZL99017 | Inbred line |  | 120 | SCHP92 | Hybrid | CML543 / U2540W |
| 27 | CML312 | Inbred line |  | 121 | SCHP93 | Hybrid | CML544 / CK21 |
| 28 | CZL0718 | Inbred line |  | 122 | SCHP94 | Hybrid | CML544 / CML442 |
| 29 | CZL0919 | Inbred line |  | 123 | SCHP95 | Hybrid | CML544 / I-38 |
| 30 | I-40 | Inbred line |  | 124 | SCHP96 | Hybrid | CML544 / I-42 |
| 31 | SCHP1 | Hybrid | CB322 / CK21 | 125 | SCHP97 | Hybrid | CML544/RO549W |
| 32 | SCHP2 | Hybrid | CB322 / I-42 | 126 | SCHP98 | Hybrid | CML547 / CML442 |
| 33 | SCHP3 | Hybrid | CB322/RO54W | 127 | SCHP99 | Hybrid | CML547 / RO549W |
| 34 | SCHP5 | Hybrid | CB323 / CK21 | 128 | SCHP100 | Hybrid | CML547 / U2540W |
| 35 | SCHP6 | Hybrid | CB323/ CML442 | 129 | SCHP101 | Hybrid | CZL068 / CK21 |
| 36 | SCHP7 | Hybrid | CB323 / I-38 | 130 | SCHP102 | Hybrid | CZL068 / I-38 |
| 37 | SCHP8 | Hybrid | CB323 / I-42 | 131 | SCHP103 | Hybrid | CZL068 / I-42 |
| 38 | SCHP9 | Hybrid | CB323/RO54W | 132 | SCHP104 | Hybrid | CZL068 / RO549W |
| 39 | SCHP10 | Hybrid | CB323/U2540W | 133 | SCHP105 | Hybrid | CZL068 / U2540W |
| 40 | SCHP11 | Hybrid | CB339/ CML442 | 134 | SCHP106 | Hybrid | I-38 / CB322 |
| 41 | SCHP12 | Hybrid | CB339 / I-38 | 135 | SCHP107 | Hybrid | I-38 / CB323 |
| 42 | SCHP13 | Hybrid | CB339/U2540W | 136 | SCHP108 | Hybrid | I-38 / CB339 |
| 43 | SCHP14 | Hybrid | CK21 / CB322 | 137 | SCHP109 | Hybrid | I-38 / CKDHL0089 |
| 44 | SCHP15 | Hybrid | CK21 / CB323 | 138 | SCHP110 | Hybrid | I-38 / CKDHL0295 |
| 45 | SCHP16 | Hybrid | CK21 / CB339 | 139 | SCHP111 | Hybrid | I-38 / CKDHL0378 |
| 46 | SCHP17 | Hybrid | CK21/CKDHL089 | 140 | SCHP112 | Hybrid | I-38 / CKDHL0470 |
| 47 | SCHP18 | Hybrid | CK21/CKDHL095 | 141 | SCHP113 | Hybrid | I-38 / CKL05022 |
| 48 | SCHP19 | Hybrid | CK21 /CKDHL0378 | 142 | SCHP114 | Hybrid | I-38 / CML202 |
| 49 | SCHP20 | Hybrid | CK21 / CML202 | 143 | SCHP115 | Hybrid | I-38 / CML216 |
| 50 | SCHP21 | Hybrid | CK21 / CML443 | 144 | SCHP116 | Hybrid | I-38 / CML443 |
| 51 | SCHP22 | Hybrid | CK21 / CML444 | 145 | SCHP117 | Hybrid | I-38 / CML444 |
| 52 | SCHP23 | Hybrid | CK21 / CML488 | 146 | SCHP118 | Hybrid | I-38 / CML488 |
| 53 | SCHP24 | Hybrid | CK21/ CML511 | 147 | SCHP119 | Hybrid | I-38 / CML511 |
| 54 | SCHP25 | Hybrid | CK21 / CML544 | 148 | SCHP120 | Hybrid | I-38 / CML543 |
| 55 | SCHP26 | Hybrid | CK21 / CML547 | 149 | SCHP121 | Hybrid | I-38 / CML544 |
| 56 | SCHP27 | Hybrid | CK21 / CZL068 | 150 | SCHP122 | Hybrid | I-38 / CML547 |
| 57 | SCHP28 | Hybrid | CKDHL0089/ CK21 | 151 | SCHP123 | Hybrid | I-38 / CZL068 |
| 58 | SCHP29 | Hybrid | CKDHL0089/CML442 | 152 | SCHP124 | Hybrid | I-42 / CB323 |
| 59 | SCHP30 | Hybrid | CKDHL0089/RO549W | 153 | SCHP125 | Hybrid | I-42 / CB339 |
| 60 | SCHP31 | Hybrid | CKDHL0089/ U2540W | 154 | SCHP126 | Hybrid | I-42 / CKDHL0295 |
| 61 | SCHP32 | Hybrid | CKDHL0089/ I-38 | 155 | SCHP127 | Hybrid | I-42 / CKL05022 |
| 62 | SCHP33 | Hybrid | CKDHL0295/ CK21 | 156 | SCHP128 | Hybrid | I-42 / CML202 |
| 63 | SCHP35 | Hybrid | CKDHL0295 / U2540W | 157 | SCHP129 | Hybrid | I-42 / CML216 |
| 64 | SCHP36 | Hybrid | CKDHL0295 / I-38 | 158 | SCHP130 | Hybrid | I-42 / CML443 |
| 65 | SCHP37 | Hybrid | CKDHL0295 / I-42 | 159 | SCHP131 | Hybrid | I-42 / CML444 |
| 66 | SCHP38 | Hybrid | CKDHL0378 / CML442 | 160 | SCHP132 | Hybrid | I-42 / CML488 |
| 67 | SCHP39 | Hybrid | CKDHL0378 / I-38 | 161 | SCHP133 | Hybrid | I-42 / CML511 |
| 68 | SCHP40 | Hybrid | CKDHL0378 / I-42 | 162 | SCHP134 | Hybrid | I-42 / CML544 |
| 69 | SCHP41 | Hybrid | CKDHL0378 / U2540W | 163 | SCHP135 | Hybrid | I-42 / CML547 |
| 70 | SCHP42 | Hybrid | CKDHL0470 / CK21 | 164 | SCHP136 | Hybrid | RO549W/CKDHL0295 |
| 71 | SCHP43 | Hybrid | CKDHL0470 / RO549W | 165 | SCHP137 | Hybrid | RO549W/CKDHL0470 |
| 72 | SCHP44 | Hybrid | CKDHL0470 / I-38 | 166 | SCHP138 | Hybrid | RO549W/CKL05022 |
| 73 | SCHP45 | Hybrid | CKDHL0470 / I-42 | 167 | SCHP139 | Hybrid | RO549W / CML216 |
| 74 | SCHP46 | Hybrid | CKDHL0470 /U2540W | 168 | SCHP140 | Hybrid | RO549W / CML444 |
| 75 | SCHP47 | Hybrid | CKL05022 / I-38 | 169 | SCHP141 | Hybrid | RO549W / CML488 |
| 76 | SCHP48 | Hybrid | CKL05022 / I-42 | 170 | SCHP142 | Hybrid | RO549W / CML511 |
| 77 | SCHP49 | Hybrid | CKL05022 / RO549W | 171 | SCHP143 | Hybrid | RO549W / CML543 |
| 78 | SCHP50 | Hybrid | CKL05022 / U2540W | 172 | SCHP144 | Hybrid | RO549W / CML544 |
| 79 | SCHP51 | Hybrid | CML202 / CK21 | 173 | SCHP145 | Hybrid | RO549W / CML547 |
| 80 | SCHP52 | Hybrid | CML202 / CML442 | 174 | SCHP146 | Hybrid | RO549W / CZL068 |
| 81 | SCHP53 | Hybrid | CML202 / I-38 | 175 | SCHP147 | Hybrid | U2540W / CB322 |
| 82 | SCHP54 | Hybrid | CML202 / I-42 | 176 | SCHP148 | Hybrid | U2540W / CB323 |
| 83 | SCHP55 | Hybrid | CML216 / CML442 | 177 | SCHP149 | Hybrid | U2540W/CKDHL0295 |
| 84 | SCHP56 | Hybrid | CML216 / I-42 | 178 | SCHP150 | Hybrid | U2540W/CKDHL0378 |
| 85 | SCHP57 | Hybrid | CML216 / RO549W | 179 | SCHP151 | Hybrid | U2540W/CKDHL0470 |
| 86 | SCHP58 | Hybrid | CML216 / U2540W | 180 | SCHP152 | Hybrid | U2540W / CKL05022 |
| 87 | SCHP59 | Hybrid | CML442 / CB322 | 181 | SCHP153 | Hybrid | U2540W / CML216 |
| 88 | SCHP60 | Hybrid | CML442 / CB323 | 182 | SCHP155 | Hybrid | U2540W / CML511 |
| 89 | SCHP61 | Hybrid | CML442 / CB339 | 183 | SCHP156 | Hybrid | U2540W / CML543 |
| 90 | SCHP62 | Hybrid | CML442/CKDHL0089 | 184 | SCHP157 | Hybrid | U2540W / CML547 |
| 91 | SCHP63 | Hybrid | CML442 / CKDHL0378 | 185 | SCHP158 | Hybrid | CML540 / CZL99017 |
| 92 | SCHP64 | Hybrid | CML442 / CML216 | 186 | SCHP159 | Hybrid | CZL0718 / CZL0919 |
| 93 | SCHP65 | Hybrid | CML442 / CML443 | 187 | SCHP160 | Hybrid | CML312 / CML443 |
| 94 | SCHP66 | Hybrid | CML442 / CML444 | 188 | SCHP161 | Hybrid | I-40 / CML312 |

**No** = genotype number
