# Supplementary material for: SNP-based assessment of genetic purity and diversity in maize hybrid breeding
Source: PLoS One. 2021 Aug 3;16(8):e0249505. doi: 10.1371/journal.pone.0249505 (PMC8330893; doi:10.1371/journal.pone.0249505)
Supplement: S2 Table — (DOCX) [file pone.0249505.s002.docx]

**S2 Table. List of 92 SNPs markers used in this study and their chromosomal** **position**

| No | SNP Name | Chrome # | Physical Position(BP) | No | SNP NAME | Chrome # | Physical Position(BP) |
| --- | --- | --- | --- | --- | --- | --- | --- |
| 1 | PZA00175_2 | 1 | 8,510,027 | 56 | PZA00214_1 | 6 | 91,704,092 |
| 2 | PZB01062_3 | 1 | 56,846,728 | 57 | PZB01658_1 | 6 | 102,953,833 |
| 3 | PHM10621_29 | 1 | 101,421,468 | 58 | lac1_3 | 6 | 120,230,802 |
| 4 | PHM1968_22 | 1 | 183,647,544 | 59 | PZA02187_1 | 6 | 139,106,115 |
| 5 | PHM12706_14 | 1 | 212,356,401 | 60 | PZA02436_1 | 6 | 149,251,173 |
| 6 | PZA00664_3 | 1 | 227,542,649 | 61 | PZA01462_1 | 6 | 155,546,716 |
| 7 | PZA02269_3 | 1 | 252,722,026 | 62 | PHM3466_69 | 6 | 167,148,384 |
| 8 | PZB01403_1 | 1 | 285,273,845 | 63 | PHM3078_12 | 7 | 5,963,009 |
| 9 | PZA02737_1 | 1 | 69(Cm) | 64 | PHM4080_15 | 7 | 20,240,404 |
| 10 | PHM13440_13 | 2 | 2,527,344 | 65 | PZA00084_2 | 7 | 43,948,264 |
| 11 | PZA03629_1 | 2 | 72 (Cm) | 66 | PZA01607_1 | 7 | 68,051,112 |
| 12 | PHM6111_5 | 2 | 21,990,814 | 67 | PZA03645_1 | 7 | 73,892,322 |
| 13 | PZA02378_7 | 2 | 35,040,818 | 68 | PZA01933_3 | 7 | 98,070,498 |
| 14 | PHM3457_6 | 2 | 62,804,122 | 69 | PZA01533_2 | 7 | 162,381,818 |
| 15 | PHM13360_13 | 2 | 107,146,579 | 70 | PZA02174_2 | 8 | 4,101,256 |
| 16 | PHM3626_3 | 2 | 125,642,617 | 71 | PHM2350_17 | 8 | 23,985,819 |
| 17 | PZA03211_6 | 2 | 148,837,605 | 72 | PZA00498_5 | 8 | 48,775,713 |
| 18 | PZA00495_5 | 2 | 170,377,814 | 73 | PZA00793_2 | 8 | 64,421,988 |
| 19 | PHM3668_12 | 2 | 195,555,350 | 74 | PHM11114_7 | 8 | 70,899,841 |
| 20 | PZA00527_10 | 2 | 216,833,071 | 75 | PHM4134_8 | 8 | 105,795,742 |
| 21 | PZD00022_5 | 2 | 233,128,511 | 76 | PZA00770_1 | 8 | 134,140,609 |
| 22 | PZA02090_1 | 3 | 4,138,512 | 77 | PZA03182_5 | 8 | 152,155,087 |
| 23 | PHM2343_25 | 3 | 27,981,649 | 78 | PHM2749_10 | 8 | 171,703,522 |
| 24 | PZA01447_1 | 3 | 53,549,251 | 79 | sh1_12 | 9 | 11,340,882 |
| 25 | PHM5502_31 | 3 | 67,284,067 | 80 | PHM5181_10 | 9 | 15,582,065 |
| 26 | PZA02742_1 | 3 | 97,441,783 | 81 | PZA01791_2 | 9 | 77,467,426 |
| 27 | PZA00413_20 | 3 | 125,192,432 | 82 | PZA01062_1 | 9 | 88,057,320 |
| 28 | PZA00667_2 | 3 | 161,516,227 | 83 | PZB01899_1 | 9 | 98,502,843 |
| 29 | PHM17210_5 | 3 | 178,229,653 | 84 | PZA02325_4 | 9 | 117,870,773 |
| 30 | PZA03154_2 | 3 | 109 (Cm) | 85 | wx1_1 | 9 | 40 (cM) |
| 31 | PZB01109_1 | 3 | 194,643,731 | 86 | PZA01715_2 | 9 | 142,948,449 |
| 32 | PZA03527_3 | 3 | 22(cM) | 87 | PHM3922_32 | 10 | 17,722,938 |
| 33 | PZA02358_1 | 4 | 11,329,241 | 88 | PHM13687_14 | 10 | 47 (cM) |
| 34 | PHM5572_19 | 4 | 35,384,118 | 89 | PZA03603_1 | 10 | 75 (cM) |
| 35 | PZA00726_10 | 4 | 60,768,063 | 90 | PHM2770_19 | 10 | 72,565,410 |
| 36 | PZA00218_1 | 4 | 78,946,415 | 91 | PZA01919_2 | 10 | 111,260,278 |
| 37 | PZA03536_1 | 4 | 107,751,353 | 92 | PZA00866_2 | 10 | 124,203,565 |
| 38 | PZA03409_1 | 4 | 128,632,208 |  |  |  |  |
| 39 | PZA01477_3 | 4 | 172,301,064 |  |  |  |  |
| 40 | PZA02779_1 | 4 | 207,114,208 |  |  |  |  |
| 41 | PZA03322_5 | 4 | 242,019,440 |  |  |  |  |
| 42 | PZA02462_1 | 5 | 6,820,571 |  |  |  |  |
| 43 | Ae1_8 | 5 | 79(cM) |  |  |  |  |
| 44 | PHM13639_13 | 5 | 152(cM) |  |  |  |  |
| 45 | PZA01427_1 | 5 | 23,135,578 |  |  |  |  |
| 46 | PZA00981_3 | 5 | 37,030,384 |  |  |  |  |
| 47 | PHM4165_14 | 5 | 65,741,535 |  |  |  |  |
| 48 | PZA00643_13 | 5 | 91,096,945 |  |  |  |  |
| 49 | PZA02164_16 | 5 | 112,179,855 |  |  |  |  |
| 50 | PHM662_27 | 5 | 135,569,668 |  |  |  |  |
| 51 | ae1_7 | 5 | 167,873,309 |  |  |  |  |
| 52 | PZA00352_23 | 5 | 191,075,557 |  |  |  |  |
| 53 | PZA02480_1 | 5 | 214,953,055 |  |  |  |  |
| 54 | PZA00440_1 | 6 | 22,404,308 |  |  |  |  |
| 55 | PZA00355_2 | 6 | 78,756,133 |  |  |  |  |
