# Supplementary material for: SNP-based assessment of genetic purity and diversity in maize hybrid breeding
Source: PLoS One. 2021 Aug 3;16(8):e0249505. doi: 10.1371/journal.pone.0249505 (PMC8330893; doi:10.1371/journal.pone.0249505)
Supplement: S3 Table — (DOCX) [file pone.0249505.s003.docx]

**S3 Table. Parent-offspring test for the evaluated 158 maize hybrids**

| Hybrid | Pedigree | % SNPs parent A | % SNPs parent B | % SNPs shared by both | % SNPs belong to neither parents |
| --- | --- | --- | --- | --- | --- |
| SCHP7 | CB323 / I-38 | 26.09 | 26.09 | 47.83 | 0.00 |
| SCHP19 | CK21 / CKDHL0378 | 20.11 | 23.91 | 55.98 | 0.00 |
| SCHP20 | CK21 / CML202 | 20.65 | 22.28 | 57.07 | 0.00 |
| SCHP28 | CKDHL0089 / CK21 | 29.35 | 26.63 | 44.02 | 0.00 |
| SCHP29 | CKDHL0089/ CML442 | 23.91 | 25.54 | 49.46 | 0.00 |
| SCHP33 | CKDHL0295/ CK21 | 23.91 | 20.11 | 55.98 | 0.00 |
| SCHP52 | CML202 / CML442 | 27.17 | 25.00 | 47.83 | 0.00 |
| SCHP60 | CML442 / CB323 | 27.72 | 25.54 | 46.74 | 0.00 |
| SCHP61 | CML442 / CB339 | 25.00 | 23.37 | 51.63 | 0.00 |
| SCHP65 | CML442 / CML443 | 24.46 | 24.46 | 48.91 | 0.00 |
| SCHP68 | CML442 / CML511 | 28.8 | 26.63 | 44.57 | 0.00 |
| SCHP73 | CML443 / CML442 | 24.46 | 25.54 | 48.91 | 0.00 |
| SCHP86 | CML511 / CML442 | 27.17 | 28.26 | 44.57 | 0.00 |
| SCHP93 | CML544 / CK21 | 27.72 | 25.54 | 45.65 | 0.00 |
| SCHP115 | I-38 / CML216 | 19.57 | 21.74 | 57.61 | 0.00 |
| SCHP133 | I-42 / CML511 | 2.17 | 29.89 | 66.85 | 0.00 |
| SCHP6 | CB323 / CML442 | 25.00 | 28.26 | 46.20 | 0.54 |
| SCHP11 | CB339 / CML442 | 22.83 | 23.91 | 51.63 | 0.54 |
| SCHP12 | CB339 / I-38 | 24.46 | 21.20 | 52.72 | 0.54 |
| SCHP27 | CK21 / CZL068 | 20.65 | 19.02 | 58.70 | 0.54 |
| SCHP36 | CKDHL0295 / I-38 | 21.20 | 17.93 | 60.33 | 0.54 |
| SCHP39 | CKDHL0378 / I-38 | 24.46 | 20.65 | 54.35 | 0.54 |
| SCHP44 | CKDHL0470 / I-38 | 26.09 | 22.28 | 50.00 | 0.54 |
| SCHP47 | CKL05022 / I-38 | 22.28 | 19.57 | 57.61 | 0.54 |
| SCHP51 | CML202 / CK21 | 21.20 | 19.57 | 56.52 | 0.54 |
| SCHP53 | CML202 / I-38 | 24.46 | 21.74 | 53.26 | 0.54 |
| SCHP64 | CML442 / CML216 | 24.46 | 23.91 | 50.00 | 0.54 |
| SCHP67 | CML442 / CML488 | 20.65 | 26.09 | 50.54 | 0.54 |
| SCHP69 | CML442 / CML544 | 25.54 | 26.63 | 47.28 | 0.54 |
| SCHP70 | CML442 / CML547 | 25.00 | 23.37 | 51.09 | 0.54 |
| SCHP71 | CML442 / CZL068 | 23.37 | 21.74 | 51.09 | 0.54 |
| SCHP81 | CML488 / CML442 | 27.17 | 22.28 | 50.00 | 0.54 |
| SCHP82 | CML488 / I-38 | 25.54 | 7.07 | 65.76 | 0.54 |
| SCHP98 | CML547 / CML442 | 22.28 | 23.91 | 51.09 | 0.54 |
| SCHP101 | CZL068 / CK21 | 16.85 | 17.39 | 54.35 | 0.54 |
| SCHP110 | I-38 / CKDHL0295 | 17.93 | 21.2 | 60.33 | 0.54 |
| SCHP111 | I-38 / CKDHL0378 | 20.65 | 24.46 | 54.35 | 0.54 |
| SCHP112 | I-38 / CKDHL0470 | 23.91 | 25.54 | 50.00 | 0.54 |
| SCHP113 | I-38 / CKL05022 | 20.11 | 21.20 | 57.07 | 0.54 |
| SCHP114 | I-38 / CML202 | 22.28 | 24.46 | 53.26 | 0.54 |
| SCHP117 | I-38 / CML444 | 26.09 | 26.09 | 46.2 | 0.54 |
| SCHP119 | I-38 / CML511 | 6.52 | 24.46 | 67.39 | 0.54 |
| SCHP123 | I-38 / CZL068 | 20.11 | 21.20 | 58.15 | 0.54 |
| SCHP160 | CML312 / CML443 | 21.74 | 25.00 | 48.37 | 0.54 |
| SCHP18 | CK21 / CKDHL0295 | 20.11 | 23.91 | 54.89 | 1.09 |
| SCHP23 | CK21 / CML488 | 19.57 | 27.72 | 51.63 | 1.09 |
| SCHP26 | CK21 / CML547 | 22.28 | 22.28 | 54.35 | 1.09 |
| SCHP32 | CKDHL0089/ I-38 | 26.09 | 26.09 | 45.65 | 1.09 |
| SCHP38 | CKDHL0378 / CML442 | 23.91 | 22.28 | 52.72 | 1.09 |
| SCHP45 | CKDHL0470 / I-42 | 28.26 | 22.83 | 47.83 | 1.09 |
| SCHP48 | CKL05022 / I-42 | 22.28 | 20.65 | 53.80 | 1.09 |
| SCHP55 | CML216 / CML442 | 24.46 | 23.91 | 50.54 | 1.09 |
| SCHP63 | CML442 / CKDHL0378 | 22.83 | 23.91 | 52.17 | 1.09 |
| SCHP74 | CML443 / I-38 | 27.17 | 26.63 | 45.11 | 1.09 |
| SCHP75 | CML443 / I-42 | 28.80 | 25.00 | 44.02 | 1.09 |
| SCHP78 | CML444 / I-38 | 22.28 | 25.00 | 51.63 | 1.09 |
| SCHP80 | CML488 / CK21 | 28.26 | 20.11 | 50.54 | 1.09 |
| SCHP90 | CML543 / I-38 | 21.74 | 23.37 | 53.8 | 1.09 |
| SCHP94 | CML544 / CML442 | 26.09 | 24.46 | 46.20 | 1.09 |
| SCHP102 | CZL068 / I-38 | 23.91 | 22.28 | 52.72 | 1.09 |
| SCHP107 | I-38 / CB323 | 26.09 | 23.91 | 46.74 | 1.09 |
| SCHP108 | I-38 / CB339 | 21.20 | 23.37 | 53.26 | 1.09 |
| SCHP109 | I-38 / CKDHL0089 | 25.00 | 26.09 | 46.74 | 1.09 |
| SCHP116 | I-38 / CML443 | 25.00 | 39.67 | 46.20 | 1.09 |
| SCHP121 | I-38 / CML544 | 23.37 | 21.20 | 54.35 | 1.09 |
| SCHP122 | I-38 / CML547 | 18.48 | 20.65 | 57.61 | 1.09 |
| SCHP124 | I-42 / CB323 | 22.28 | 23.91 | 52.72 | 1.09 |
| SCHP125 | I-42 / CB339 | 26.09 | 26.63 | 46.20 | 1.09 |
| SCHP127 | I-42 / CKL05022 | 17.39 | 21.20 | 59.24 | 1.09 |
| SCHP158 | CML540 / CZL99017 | 22.28 | 22.28 | 52.17 | 1.09 |
| SCHP15 | CK21 / CB323 | 27.17 | 26.09 | 44.02 | 1.63 |
| SCHP17 | CK21 / CKDHL0089 | 25.54 | 28.26 | 43.48 | 1.63 |
| SCHP21 | CK21 / CML443 | 20.65 | 26.63 | 51.09 | 1.63 |
| SCHP24 | CK21/ CML511 | 21.74 | 21.20 | 55.43 | 1.63 |
| SCHP40 | CKDHL0378 / I-42 | 23.91 | 17.39 | 55.98 | 1.63 |
| SCHP59 | CML442 / CB322 | 30.43 | 22.83 | 45.11 | 1.63 |
| SCHP66 | CML442 / CML444 | 28.26 | 22.83 | 46.20 | 1.63 |
| SCHP72 | CML443 / CK21 | 25.00 | 22.83 | 50.54 | 1.63 |
| SCHP87 | CML511 / I-38 | 22.83 | 22.28 | 53.26 | 1.63 |
| SCHP95 | CML544 / I-38 | 21.74 | 18.48 | 57.07 | 1.63 |
| SCHP103 | CZL068 / I-42 | 23.91 | 19.02 | 52.17 | 1.63 |
| SCHP126 | I-42 / CKDHL0295 | 20.11 | 28.80 | 49.46 | 1.63 |
| SCHP130 | I-42 / CML443 | 16.85 | 23.37 | 33.15 | 1.63 |
| SCHP131 | I-42 / CML444 | 24.46 | 31.52 | 42.39 | 1.63 |
| SCHP5 | CB323 / CK21 | 25.54 | 28.26 | 44.02 | 2.17 |
| SCHP16 | CK21 / CB339 | 23.37 | 23.91 | 50.54 | 2.17 |
| SCHP25 | CK21 / CML544 | 27.72 | 23.91 | 44.02 | 2.17 |
| SCHP30 | CKDHL0089 / RO549W | 40.22 | 20.11 | 37.5 | 2.17 |
| SCHP42 | CKDHL0470 / CK21 | 28.80 | 27.17 | 39.67 | 2.17 |
| SCHP85 | CML511 / CK21 | 21.20 | 20.65 | 55.98 | 2.17 |
| SCHP120 | I-38 / CML543 | 21.74 | 21.20 | 52.72 | 2.17 |
| SCHP129 | I-42 / CML216 | 21.74 | 30.43 | 45.65 | 2.17 |
| SCHP135 | I-42 / CML547 | 14.67 | 21.20 | 60.87 | 2.17 |
| SCHP8 | CB323 / I-42 | 29.89 | 23.91 | 43.48 | 2.72 |
| SCHP37 | CKDHL0295 / I-42 | 22.28 | 15.22 | 58.70 | 2.72 |
| SCHP56 | CML216 / I-42 | 27.72 | 20.11 | 49.46 | 2.72 |
| SCHP96 | CML544 / I-42 | 23.37 | 17.93 | 55.98 | 2.72 |
| SCHP54 | CML202 / I-42 | 30.43 | 21.20 | 45.11 | 3.26 |
| SCHP99 | CML547 / RO549W | 34.24 | 19.02 | 40.22 | 3.26 |
| SCHP106 | I-38 / CB322 | 23.37 | 20.65 | 50.54 | 3.26 |
| SCHP14 | CK21 / CB322 | 22.83 | 24.46 | 48.91 | 3.80 |
| SCHP128 | I-42 / CML202 | 17.39 | 27.72 | 50.00 | 3.80 |
| SCHP134 | I-42 / CML544 | 17.39 | 22.83 | 54.89 | 3.80 |
| SCHP136 | RO549W / CKDHL0295 | 20.11 | 30.98 | 32.07 | 3.80 |
| SCHP144 | RO549W / CML544 | 23.91 | 32.61 | 35.33 | 3.80 |
| SCHP57 | CML216 / RO549W | 25.54 | 19.57 | 49.46 | 4.35 |
| SCHP83 | CML488 / RO549W | 29.89 | 23.37 | 42.39 | 4.35 |
| SCHP161 | I-40 / CML312 | 20.65 | 23.37 | 49.46 | 4.35 |
| SCHP1 | CB322 / CK21 | 25.00 | 22.28 | 47.83 | 4.89 |
| SCHP2 | CB322 / I-42 | 23.91 | 21.20 | 50.00 | 4.89 |
| SCHP88 | CML511 / I-42 | 22.83 | 18.48 | 50.54 | 4.89 |
| SCHP145 | RO549W / CML547 | 21.20 | 34.24 | 39.67 | 4.89 |
| SCHP89 | CML511 / RO549W | 28.80 | 21.74 | 44.02 | 5.43 |
| SCHP97 | CML544 / RO549W | 35.87 | 23.37 | 35.33 | 5.43 |
| SCHP138 | RO549W / CKL05022 | 17.93 | 26.63 | 48.91 | 5.43 |
| SCHP142 | RO549W / CML511 | 20.11 | 29.35 | 44.02 | 5.43 |
| SCHP49 | CKL05022 / RO549W | 28.80 | 16.85 | 48.37 | 5.98 |
| SCHP137 | RO549W / CKDHL0470 | 18.48 | 32.61 | 41.85 | 5.98 |
| SCHP9 | CB323 / RO549W | 23.37 | 23.91 | 45.11 | 6.52 |
| SCHP43 | CKDHL0470 / RO549W | 36.41 | 13.59 | 42.39 | 6.52 |
| SCHP77 | CML444 / CML442 | 13.04 | 38.04 | 40.22 | 6.52 |
| SCHP92 | CML543 / U2540W | 34.78 | 10.33 | 47.28 | 6.52 |
| SCHP146 | RO549W / CZL068 | 14.67 | 32.07 | 45.65 | 6.52 |
| SCHP152 | U2540W / CKL05022 | 16.85 | 33.15 | 36.96 | 6.52 |
| SCHP50 | CKL05022 / U2540W | 33.70 | 18.48 | 40.22 | 7.61 |
| SCHP104 | CZL068 / RO549W | 32.61 | 16.30 | 40.22 | 7.61 |
| SCHP150 | U2540W / CKDHL0378 | 15.22 | 35.33 | 40.76 | 7.61 |
| SCHP159 | CZL0718 / CZL0919 | 21.74 | 13.04 | 54.35 | 7.61 |
| SCHP3 | CB322 / RO549W | 23.37 | 20.11 | 48.37 | 8.15 |
| SCHP10 | CB323 / U2540W | 35.33 | 21.20 | 34.24 | 8.15 |
| SCHP13 | CB339 / U2540W | 35.87 | 11.96 | 42.93 | 8.15 |
| SCHP35 | CKDHL0295 / U2540W | 35.33 | 11.96 | 44.57 | 8.15 |
| SCHP58 | CML216 / U2540W | 33.15 | 18.48 | 39.13 | 8.15 |
| SCHP147 | U2540W / CB322 | 18.48 | 36.96 | 36.41 | 8.15 |
| SCHP46 | CKDHL0470 / U2540W | 30.43 | 12.50 | 47.28 | 8.70 |
| SCHP91 | CML543 / RO549W | 27.72 | 15.22 | 48.37 | 8.70 |
| SCHP118 | I-38 / CML488 | 33.15 | 15.22 | 41.85 | 8.70 |
| SCHP139 | RO549W / CML216 | 17.39 | 31.52 | 42.39 | 8.70 |
| SCHP141 | RO549W / CML488 | 15.76 | 35.87 | 39.67 | 8.70 |
| SCHP153 | U2540W / CML216 | 15.76 | 35.87 | 37.50 | 8.70 |
| SCHP41 | CKDHL0378 / U2540W | 33.15 | 16.30 | 39.13 | 9.24 |
| SCHP143 | RO549W / CML543 | 13.04 | 27.72 | 47.83 | 9.24 |
| SCHP149 | U2540W / CKDHL0295 | 15.76 | 30.43 | 41.30 | 9.24 |
| SCHP156 | U2540W / CML543 | 17.93 | 33.70 | 39.13 | 9.24 |
| SCHP157 | U2540W / CML547 | 14.13 | 30.43 | 42.93 | 9.24 |
| SCHP31 | CKDHL0089/ U2540W | 25.54 | 14.13 | 49.46 | 9.78 |
| SCHP151 | U2540W / CKDHL0470 | 17.39 | 26.63 | 45.11 | 9.78 |
| SCHP76 | CML444 / CK21 | 17.39 | 29.89 | 42.39 | 10.33 |
| SCHP100 | CML547 / U2540W | 23.37 | 22.83 | 38.04 | 10.33 |
| SCHP148 | U2540W / CB323 | 17.93 | 39.13 | 32.61 | 10.33 |
| SCHP62 | CML442 / CKDHL0089 | 39.67 | 11.96 | 37.5 | 10.87 |
| SCHP155 | U2540W / CML511 | 11.96 | 23.91 | 50.00 | 10.87 |
| SCHP105 | CZL068 / U2540W | 27.72 | 26.09 | 32.61 | 11.41 |
| SCHP84 | CML488 / U2540W | 30.43 | 11.96 | 45.11 | 12.50 |
| SCHP132 | I-42 / CML488 | 26.09 | 15.76 | 40.76 | 13.04 |
| SCHP22 | CK21 / CML444 | 30.98 | 13.59 | 41.85 | 13.59 |
| SCHP79 | CML444 / I-42 | 17.93 | 27.17 | 36.41 | 18.48 |
| SCHP140 | RO549W / CML444 | 18.48 | 19.57 | 42.39 | 19.57 |
| Mean |  | 23.73 | 23.48 | 48.05 | 3.60 |
| SE |  | 0.46 | 0.45 | 0.55 | 0.31 |
